# In Vitro Propagation, Genetic Assessment, and Medium-Term Conservation of the Coastal Endangered Species *Tetraclinis articulata* (Vahl) Masters (Cupressaceae) from Adult Trees

**DOI:** 10.3390/plants11020187

**Published:** 2022-01-11

**Authors:** Jorge Juan-Vicedo, Francisco Serrano-Martínez, Miriam Cano-Castillo, José Luis Casas

**Affiliations:** 1 Instituto de Investigación en Medio Ambiente y Ciencia Marina IMEDMAR, Universidad Católica de Valencia, Carrer Guillem de Castro, 94, 46001 València, Spain; 2 Research Institute CIBIO (Centro Iberoamericano de la Biodiversidad), Science Park, University of Alicante, Ctra. San Vicente del Raspeig s/n, 03690 Alicante, Spain; f.serrano@ua.es (F.S.-M.); miriam.cano@ua.es (M.C.-C.); jl.casas@ua.es (J.L.C.)

**Keywords:** conifer, endangered plant, genetic fidelity, micropropagation, plant growth regulator, slow growth, *Tetraclinis articulata*, tissue culture

## Abstract

*Tetraclinis articulata* (Vahl) Masters is an endangered tree growing in coastal and arid environments that is widely exploited by the timber and resin industry, among other applications. In this context, the use of in vitro techniques is highly encouraged for its propagation. We present a protocol for micropropagation using twigs from adult trees as a source of explants. The Schenk and Hildebrandt basal medium (SH) supplemented with 30 g L^−1^ sucrose, 6.5 g L^−1^ plant agar, 4.0 mg L^−1^ 6-benzyladenine (BA), and 0.05 mg L^−1^ 1-naphthaleneacetic acid (NAA) provided the optimum multiplication rate (90.48 ± 9.52 explants with basal shoots and 2.58 ± 0.29 basal shoots per explant). Application of activated charcoal (AC) or ½ Knop solution in a liquid overlay produced significantly longer shoots. Supplementation of solid media with indole-3-butyric acid (IBA) or NAA gave low rooting percentages (<17%). Addition of 0.9 g L^−1^ AC improved rooting (40%) but rooting performance was optimal (66.7%) after a pulse treatment consisting of 4 h immersion in liquid SH medium without growth regulators, followed by 8 weeks of cultivation. Rooted microplants were successfully acclimatized (93.33%) in a peat moss and vermiculite mixture (1:1 *v*/*v* ratio). The genetic stability of the in vitro regenerated plantlets was confirmed using the randomly amplified polymorphic DNA (RAPD) technique. Explant survival and growth remained higher than 90% after 28 weeks of cold storage at both 4 °C and 10 °C. The protocol presented here allows for largescale *T. articulata* production and could be applied for both ex situ conservation strategies and industrial purposes.

## 1. Introduction

*Tetraclinis articulata* (Vahl) Masters (Cupressaceae) is an evergreen coniferous tree distributed discontinuously in a limited area along the South-Western Mediterranean basin [1]. In Europe, it can be punctually found in littoral environments in Malta and in the Iberian Peninsula, in the latter restricted to a single population in the coast of Cartagena. In North Africa, it is more abundant, and also forms inland forests in arid and semiarid environments [2].

*T. articulata* has a long history of usage due to its wood quality, durability, and the ornamental value of the tree [2,3,4,5]. Additionally, it has been traditionally exploited as a source of sandarac resin [6,7]. Medicinally, it is used in folk medicine against diverse pathologies of the digestive system, in dermocosmetics, and to treat allergy in Eastern Morocco [8]. Also, it is used as an antidiabetic [9] and to treat flu, coughs, or rheumatism [10]. Some works on the biological activities of its resins, essential oil, and extracts have revealed the antioxidant, antibacterial, and cytotoxic effects due to its richness in diterpenoids, among other compounds [11,12].

In addition, the intense demand by the craft industry for timbers has led to a degradation of *T. articulata* forests, thus increasing fragmentation and isolation of the natural populations, and to the current concern on this species as recently reviewed by Fidah et al. [5]. Other threats documented for *T. articulata* include the strong competition with Aleppo pine (*Pinus halepensis* Mill.) for the habitat and resources [13], and overgrazing [2]. Therefore, it was firstly included in a priority natural habitat according to the Council Directive 92/43/EEC. Its increasing conservation concern is evidenced by its recent inclusion in checklists of threatened flora. For instance, it was recently listed as endangered at the European level by the International Union of the Nature Conservation (IUCN) [14] and at national level, it is listed as vulnerable in Spain [15].

Although seed germination seems not to be a limiting factor for the plant establishment in natural environments and greenhouse production [2,16,17], the natural viability of *T. articulata* seeds rapidly decreases [2]. In addition, the time required to obtain transplantable plantlets starting from seeds normally takes a few years [2]. Therefore, it is interesting to apply the in vitro culture techniques for *T. articulata* largescale propagation. Micropropagation of tree species is generally recommended by using explants from juvenile organs such as immature embryos or young seedlings [18]. This is particularly important in conifers [19,20] due to the cloning difficulties that they present in comparison to the angiosperms [21]. In spite of these limitations, the use of adult-tree explants for the micropropagation of conifers is also reported [22,23], sometimes after a series of grafting treatments prior to initiation of in vitro cultures [24], because the use of aged donor plants is related to a faster obtaining of mature plantlets with economical value [25]. Previous works using in vitro techniques have been performed in the attempt to produce *T. articulata* plants: micropropagation using one-year-old seedling explants was firstly performed by Morte et al. [26]. As in many other conifers [27], micropropagation of this species showed some constraints during the rooting, and acclimatization stages [26]. Furthermore, the study of the effects of arbuscular mycorrhizal fungus *Glomus fasciculatus* inoculation showed a significant improvement in survival rates during the acclimatization stage [28]. Unfortunately, none of the previous works on this species focused on the genetic stability of the regenerated plants. The study of somaclonal variation within regenerants is very important to produce true-to-type plants in plant genetic resource conservation, natural population restoration, and commercial exploitation (for wood) of the plants [29,30,31].

Finally, as a result of the ex situ conservation interests of this species, Serrano-Martinez and Casas [32] developed a cryopreservation protocol for *T. articulata* shoot tips by cold acclimatization prior to vitrification. However, these authors pointed out the difficulties of applying this technique due to the strong sensitivity of the tissues for the cryopreservation procedures applied. In this regard, the medium-term conservation of microplants at low temperatures (slow-growth storage) may be an efficient alternative for the cryopreservation in ex situ conservation, as has been revealed for other trees [33,34,35].

Therefore, this is the first work aimed to develop a complete protocol for the optimal micropropagation of *T. articulata* explants from adult individuals. The explants were sterilized and the in vitro produced shoots were elongated, rooted, acclimatized, and tested for their survival at low temperature conservation. Additionally, the genetic stability of the in vitro regenerated plants was assessed using randomly amplified polymorphic DNA (RAPD).

## 2. Results

### 2.1. Sterilization of the Plant Material and Culture Initiation

**Figure 1:**
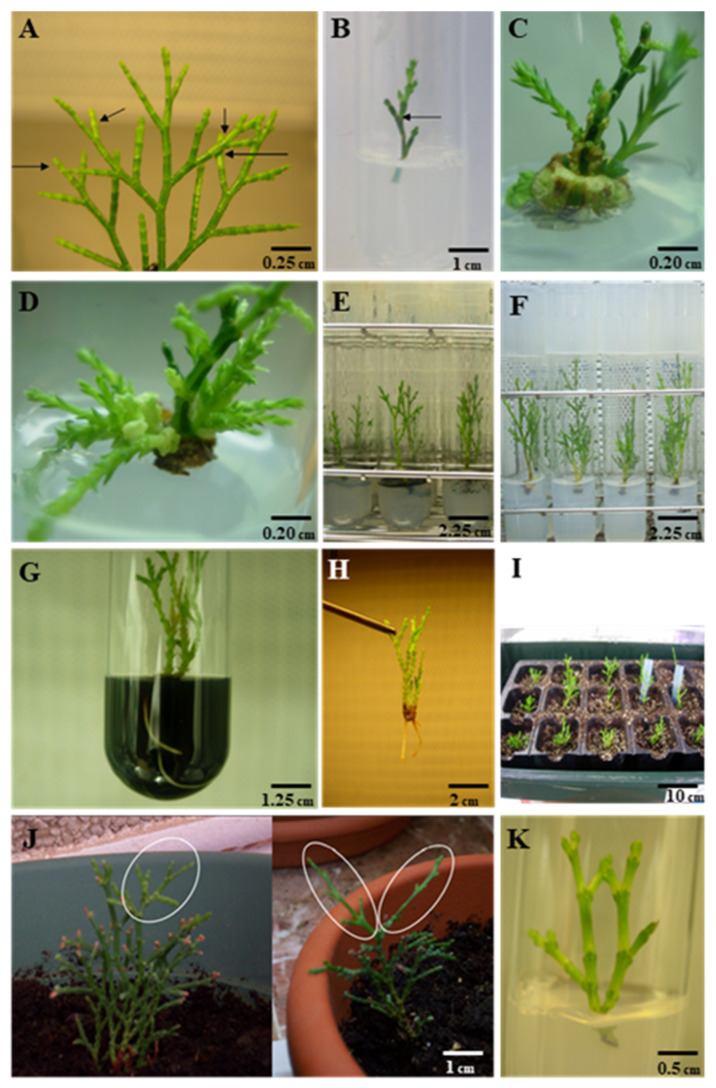
*Tetraclinis articulata* (Vahl) Masters in vitro propagation through apical shoots from adult trees. (**A**) Branches of growing individuals used as starting materials. Arrows indicate the buds taken as explants for in vitro culture initiation. (**B**) Initiated explants showing signs of growth restoration after the sterilization scheme applied (**C**) Morphogenic response of *T. articulata* explants cultured in vitro in Schenk and Hildebrandt (1972) (SH) medium supplemented with 30 g L^−1^ sucrose and 0.1 mg L^−1^ thidiazuron (TDZ): newly formed shoots and callus formation from the base of the explant. (**D**) Newly formed basal shoots under in vitro cultivation in SH medium supplemented with 30 g L^−1^ sucrose, and 1.0 mg L^−1^ 6-benzylaminopurine (BAP) + 0.05 mg L^−1^ 1-naphthaleneacetic acid (NAA) at the 12th week of culture. (**E**) Elongated explants in a double-phase system composed by SH (solid phase), and an overlay composed by half-strength Knop solution (Knop, 1865) + 50 g L^−1^ sucrose + 6 g L^−1^ activated charcoal (AC) after 8 weeks of culture. (**F**) Elongated explants in a double-phase system composed by a solid phase of SH medium and an overlay composed by ½ Knop solution + 60 g L^−1^ sucrose after 8 weeks of culture. (**G**) Root production in medium SH + 30 g L^−1^ sucrose + 0.9 g L^−1^ AC. (**H**) Rooted explants treated with an immersion of 100 mg L^−1^ indole-3-acetic acid (IAA) for one hour. (**I**) *T. articulata*-rooted microplants planted in a mixture of peat moss:vermiculite (1:1). (**J**) Regenerated and acclimatized *T. articulata* plants after six months of culture in outdoor conditions. Newly formed branches during this period are highlighted. (**K**) Regrowth of *T. articulata* explants after 28 weeks of storage at low temperatures.

The sterilization protocol applied here resulted in approximately 90% of uncontaminated explants for all trials performed (90 ± 1.14). Most of these explants were considered to be initiated as they showed signs of growth restoration without macroscopic signs of morphological and physiological abnormalities (callus, oxidative browning, chlorosis, etc.) after three weeks of culture (Figure 1B) at 25 ± 1 °C under low-active radiation conditions (0.7 μmol m^−2^ s^−1^). Then, initiated explants were placed at 42 μmol m^−2^ s^−1^ in a 16 h photoperiod, and a selected plantlet was micropropagated in order to obtain a stock of explants for further experiments.

### 2.2. Growth Regulator Effects on Multiplication

In the first experiment, all cytokinins produced statistically significant shorter shoots in comparison to the controls at the 6th and 12th weeks of culture in an almost dose-dependent manner, with the exception of thidiazuron (TDZ) at a concentration of 0.025 mg L^−1^ that did not differ from the untreated controls (Table 1). However, the tested combinations of cytokinins promoted the basal shoot formation in a non-dose dependent manner at the 6th and 12th weeks of culture (Table 1 and Figure 1C). The statistical analysis showed that 1.0 mg L^−1^ of 6-benzylaminopurine (BAP) resulted, significantly (*p* < 0.0001), in highest percentages of explants with basal shoot formation after 12 weeks of in vitro cultivation (79.85 ± 5.32%), as well as in the highest number of newly formed basal shoots per explants (2.60 ± 0.43). The lowest percentage of explants with basal shoots and basal shoots formed per explant were recorded in media supplemented with 0.5 and 1.0 mg L^−1^ kinetin (KIN) at the 6th and 12th week of culture, as well as with 0.25 mg L^−1^ TDZ at the 12th week of culture, all them not statistically different to the controls without plant growth regulators (PGRs).

In the second experiment using eight concentrations of cytokinin and auxin (Table 2), combinations of 0.5–4.0 mg L^−1^ BAP plus 0.5 mg L^−1^ 1-naphthaleneacetic acid (NAA) yielded the highest percentage of explants with basal shoots ranging from 75.6 to 90.5% by the 12th week of culture (Figure 1D). In contrast, the lowest shoot length increment for this experiment was obtained in medium supplemented with 4.0 mg L^−1^ BAP plus 0.5 mg L^−1^ NAA at the 6th (0.19 ± 0.03 cm) and 12th (0.38 ± 0.07 cm) weeks of culture.

### 2.3. Double-Phase Culture System (DPS) Effects on Shoot Elongation

**Figure 2:**
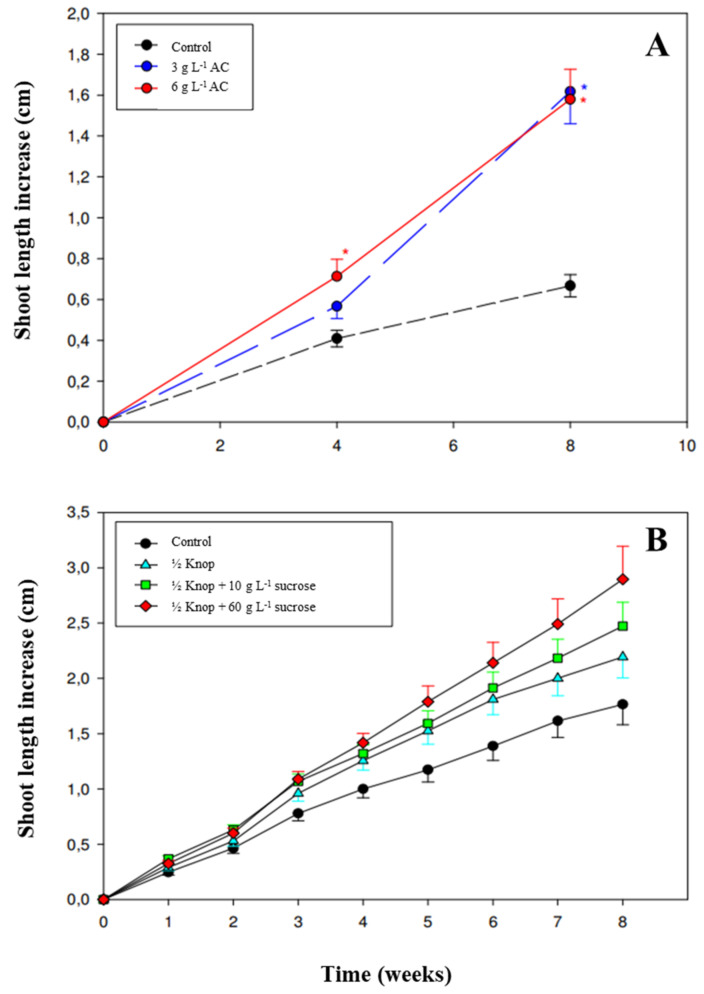
Effect of a Double-Phase Culture System (DPS) on shoot length of *Tetraclinis articulata* (Vahl) Masters explants. (**A**) The effect of two concentrations (3 and 6 g L^−1^) of activated charcoal (AC) contained in a liquid overlay was tested in a first experiment. (**B**) The effect of ½ Knop solution, and two concentrations of sucrose (10 and 60 g L^−1^) present in the overlay was tested in a second experiment. * Indicates significant differences at 5% in comparison to control as revealed by Duncan’s multiple range test. Data are presented as means ± SD.

The experiments using a DPS significantly increased the shoot elongation *T. articulata* explants in comparison to the controls. Shoots cultured with a liquid supplement containing 6 g L^−1^ activated charcoal (AC) produced higher shoots at the 4th week of culture (Figure 1E). Extending the cultivation period up to 8 weeks of shoot elongation was favored at both concentrations of AC tested (3 and 6 g L^−1^) as the height increase was significantly higher than controls, although it did not differ among them (Figure 2A).

After 8 weeks of culture, the overlays containing ½ Knop solution at both sucrose concentrations of 10 and 60 g L^−1^ significantly increased explant length in comparison to the control and the application of solely the Knop solution (*p* < 0.01). Differences found between the two concentrations of sucrose were not statistically different at the 5% level. However, the mixture containing 60 g L^−1^ showed a faster response as it increased explant length visibly since the fourth week of cultivation (Figure 1B and Figure 2B).

### 2.4. Auxin Effects on Root Development

Experiment 1. The highest rooting percentage was obtained in shoots cultured in media containing 0.5 mg L^−1^ of indole-3-butyric acid (IBA), with 16.7% rooting after 8 weeks of cultivation. Percentages of 8.3% rooted explants were obtained in media containing solely 0.5 mg L^−1^ of NAA or combinations of 0.25 mg L^−1^ NAA plus 0.5 mg L^−1^ IBA. None of the explants produced any sign of root growth in medium lacking PGRs (controls).

**Figure 3:**
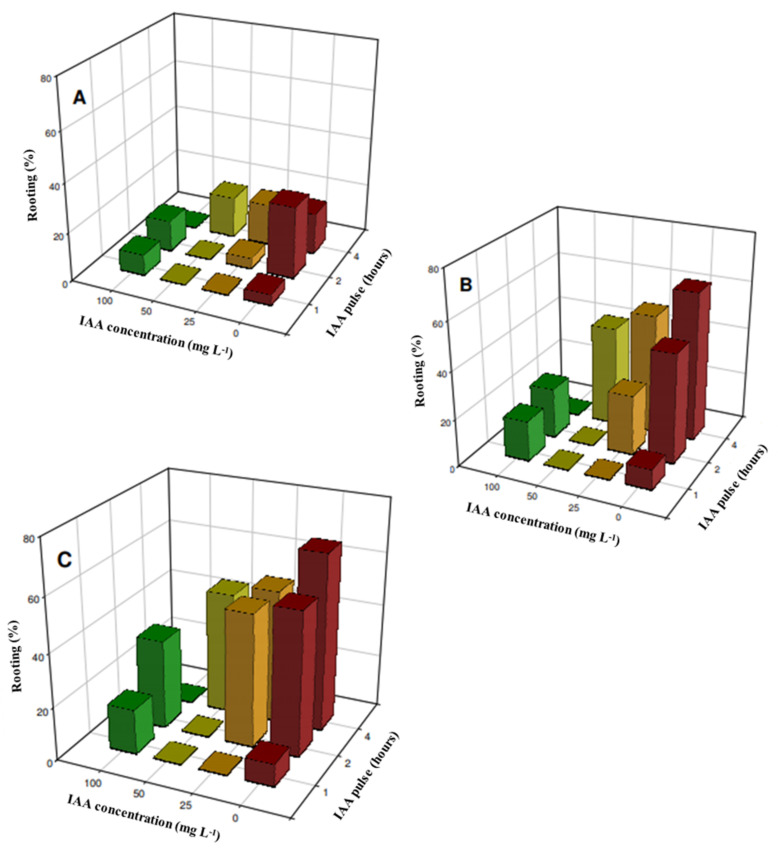
Pulse treatments performed to test the effects on rooting of *Tetraclinis articulata* (Vahl) Masters shoots cultured in vitro. Four concentrations (0, 25, 50, and 100 mg L^−1^) of IAA and three dipping times (1, 2, and 4 h) in liquid medium composed by SH salts, vitamins, and 250 mg of 2-(N-morpholino)ethanesulfonic acid (MES) were tested in dark conditions. After the pulse treatment, explants were transferred to SH basal medium supplemented with 30 g L^−1^ sucrose, 6.6 g L^−1^ agar, and 0.5 g L^−1^ AC. Rooting percentage was measured (**A**), 6 (**B**), and 8 (**C**) weeks of culture.

Experiment 2. The highest rooting percentage (66.7%) was obtained at 4 h immersion in liquid SH medium plus 250 mg 2-(N-morpholino)ethanesulfonic acid (MES) without indole-3-acetic acid (IAA; control treatment) after 8 weeks of cultivation on SH basal salts plus 30 g L^−1^ sucrose, 6.6 g L^−1^ agar, and 0.5 g L^−1^ AC (*p* < 0.01). Although the application of IAA at the highest concentration of 100 mg L^−1^ improved rooting in 1 h pulses (Figure 1H and Figure 3), this increasing was not statistically different when compared to the controls (*p* > 0.05). In some cases, the presence of IAA even decreased rooting performance in a dose-dependent manner (Figure 3).

### 2.5. Culture Media Effects on Root Development

Eight compositions of solid culture media based in SH basal medium were tested for the rooting performance and rooting percentages were recorded after 7, 8, and 10 weeks of cultivation. The highest rooting percentage (40.0%) was recorded in treatment consisting of full-strength SH basal medium plus 30 g/L sucrose and 0.9 g/L AC (Figure 1G) after 10 weeks of cultivation (*p* < 0.0001), followed by the medium composed by full-strength SH plus 15 g/L sucrose (36%). In general, culture media supplemented with AC gave higher rooting percentages than those not containing AC. In addition, media based on half-strength SH basal medium gave lower rooting percentages at all culture periods tested (*p* < 0.01).

### 2.6. Extraction of Genomic DNA and RAPD Analysis

**Figure 4:**
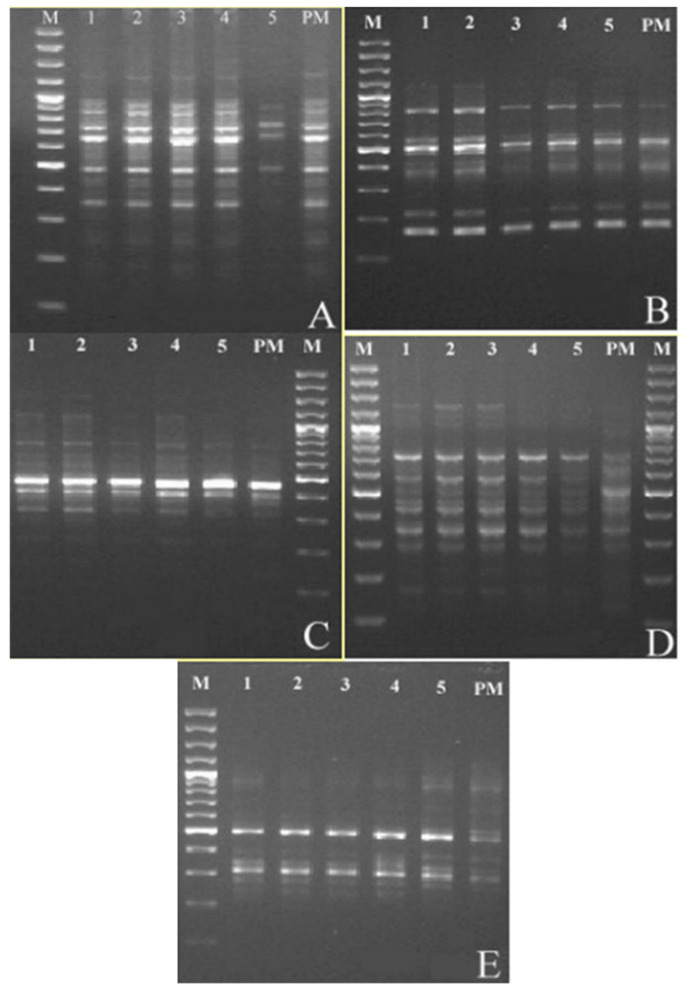
Randomly amplified polymorphic DNA profiles obtained by PCR amplification of *Tetraclinis articulata* (Vahl) Masters plant materials. M: molecular markers; 1–5: in vitro regenerated *T. articulata* plants; and PM: mother plant. Primers employed: 16 (**A**); 21 (**B**); 26 (**C**); 27 (**D**); and 29 (**E**).

Genetic stability of the micropropagated materials from natural populations were performed using genomic DNA from both the in vitro regenerated *T. articulata* plantlets and mother plant as control by means of RAPD markers. Thirty-one RAPD primers generated 29 scorable bands that ranged from 150 to 1700 bp (Table 3). The fingerprinting profiles of the *T. articulata* micropropagated plantlets using these RAPD markers yielded distinct and reproducible amplified products (Figure 4).

### 2.7. Acclimatization

Thirty *T. articulata* plantlets taken from the optimized rooting experiments showing well-formed roots after 15 weeks of culture were planted in a sterilized mixture of peat moss and vermiculite in a 1:1 (*v*/*v*) ratio in plastic trays (Figure 1I) covered with a lid. On average, twenty-eight (93.33%) of the plantlets survived over six months of under ex vitro culture conditions (Figure 1J).

### 2.8. Medium Term Storage of In Vitro Explants

**Figure 5:**
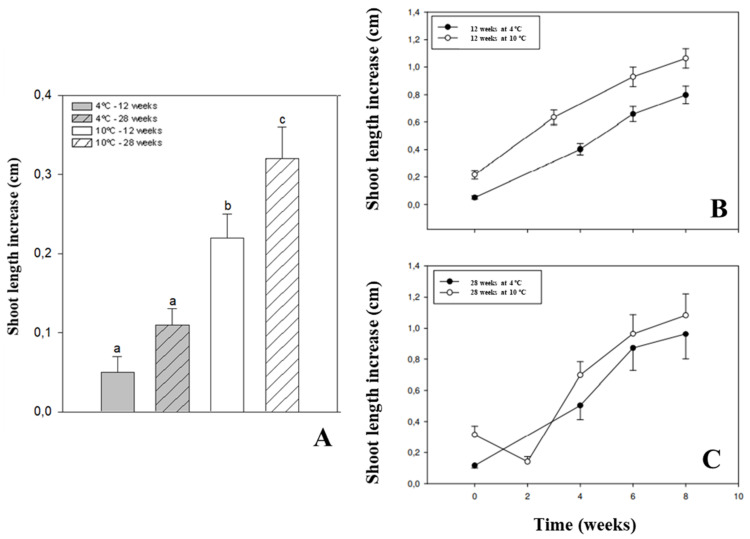
Medium-term conservation experiments for *Tetraclinis articulata* (Vahl) Masters in vitro cultured explants. The effect of two temperatures (4 °C and 10 °C) and two storage times (12 and 28 weeks) in dark conditions (stage 1) was evaluated on explants’ length increase, monitored during 8 weeks of culture onto fresh SH medium at 25 ± 1 °C under an active radiation of 42 μmol m^−2^ s^−1^ in a 16 h photoperiod (stage 2). The length increase was calculated according to the equation ∆L = FL − IL, where FL is the explant length monitored during each week of stage 2 and IL the explant length before the cold exposure (stage 1). (**A**) Overall length increase of the explants after the cold exposure (stage 1). (**B**) The evolution of the shoot length increase in explants cultured for 8 weeks after a 12-week or (**C**) 28-week periods of cold storage at 4 °C and 10 °C was also calculated. Data are presented as means ± standard errors (bars). Means with different letters are significantly different, followed by the T-test at the 5% level.

After the cold storage, all explants (100%) maintained at 10 °C for 12 weeks survived, and those treated with 4 °C showed 97.9% of survival. These percentages decreased after 28 weeks of cold storage but explant survival remained always higher than 90% for both temperatures. In terms of growth, the storage temperature significantly affected the length increase for both periods tested. Explants stored at 10 °C for 28 weeks showed the highest increase after the period of storage when compared to the explants stored for 12 weeks and explants disposed at 4 °C either for 12, or 28 weeks (stage 1; Figure 5A). After transference of the explants into stage 2, the growth of the explants for 8 weeks within in vitro culture was significantly higher in explants previously stored at 10 °C in comparison to those stored at 4 °C for the 12-week period of cold storage (Figure 5B). However, extending the cold storage period up to 28 weeks did not produce significant differences in explant regrowth for the two temperatures assayed (Figure 1K and Figure 5C).

## 3. Discussion

The sterilization protocol applied here resulted in higher than 90% of uncontaminated explants showing signs of growth restoration after three weeks of culture. Lower percentages of uncontaminated explants of 50% were obtained for one-year-old seedling explants in [26]. These authors applied a disinfestation procedure including sodium hypochlorite at 30% (also for 15 min), followed by washing in 10% hydrogen peroxide (for 5 to 8 s), and finally an immersion in 80% ethanol for 10 s. Since the seedling tissues are generally considered as more sensitive than other tissues taken from adult specimens, we think that the procedure applied by the above-mentioned authors, that included more sterilizing agents and at higher concentrations in comparison to our procedure, could be too severe for seedling explants’ survival. Hence, the protocol applied here resulted in an optimal in vitro establishment of *T. articulata* explants taken from adult trees.

The results obtained in the present study showed different optimal values for the plant developmental parameters studied at different combinations of PGRs (Table 2). For instance, the combinations of 0.5 mg L^−1^ BAP plus 0.05 mg L^−1^ NAA and 4.0 mg L^−1^ BAP plus 0.05 mg L^−1^ NAA showed the highest percentages of explants that developed basal shoots after 12 weeks of culture (84.29 ± 8.81 and 90.48 ± 9.52, respectively). Additionally, combinations of 1.0 mg L^−1^ BAP plus 0.05 mg L^−1^ NAA, and 0.5 mg L^−1^ BAP or 4.0 mg L^−1^ plus 0.05 mg L^−1^ NAA showed the highest number of newly formed basal shoots per explant after 12 weeks of culture (3.04 ± 0.25 and 2.40 ± 0.31; Table 2). Often, the criterion to select the optimal PGR mixture, and concentration for in vitro multiplication is based on the number of new shoots produced per explant. However, in some cases, there are other important features to take into account in order to identify what combination and concentration of PGRs, offers the highest multiplication potential. For instance, shoot length increment, along with the subsequent number of new nodes generated per shoot, can be even more important than solely the newly produced shoots per explant for in vitro multiplication, as described for *Sideritis leucantha* Cav. subsp. *leucantha* in [36] or *Phlomis aurea* Decne. in [37]. In the present work, both the percentage of explants that developed basal shoots, along with the number of newly formed shoots per each explant (Figure 1D), allowed for the highest number of new explants that can be obtained for further passages in vitro. Therefore, the PGRs combination of 4.0 mg L^−1^ BAP plus 0.05 mg L^−1^ NAA can be regarded as the optimal one for the in vitro multiplication of *T. articulata* (Table 2). In light of our results, BAP alone and especially in combination with NAA gave a significantly higher multiplication performance in comparison to the other cytokinins tested (Table 1) or the combinations including KIN plus NAA (Table 2). BAP is the most commonly used cytokinin in plant tissue culture [38]. It has shown to efficiently promote in vitro axillary shoot multiplication (often in combination with low amounts of auxins such as NAA) in many plant groups as recently published in Amaryllidaceae [39,40], orchids [41] as well as in conifers [42]. In the previous study performed on *T. articulata* by Morte et al. [26], BAP also showed the best results on multiplication at concentrations of 2.2 μM (≈0.5 mg L^−1^) alone or in combination with 0.054 (≈0.01 mg L^−1^) or 0.27 µM (≈0.05 mg L^−1^) NAA. Although a similar trend was observed in our study, the optimal results were obtained at higher BAP concentrations (Table 1 and Table 2). This could be explained because in [26], 1-year old seedling explants for the multiplication experiments were employed, whereas in the present study, shoot tips from mature trees were used. The organ type from which explants are taken, its developmental stage, and the endogenous level of PGRs can have influence on shoot induction ability under in vitro culture conditions [43,44,45]. Most likely, mature buds are less active than seedling explants and higher amounts of exogenous PGRs can be required to trigger cell differentiation and subsequent organogenesis in mature buds. In this regard, Zhang et al. [46] showed a reverse of maturation by reactivation of the fascicle meristem in mature buds of *Pinus radiata* D. Don treated in vitro with BAP. Additionally, young explants taken from seedlings in *Cannabis sativa* L. [47] or *Passiflora setacea* D. C. [48] have shown a high rate of shoot organogenesis when cultured in vitro on PGRs-free medium, which indicates that young tissues may even not require the application of exogenous PGRs to grow and this is most likely related to the presence of higher endogenous levels of PGRs. Similar findings are discussed in [25] for *Pinus* species. Therefore, the differences in the developmental stage of the explants used might explain the differences found when comparing our results to those obtained by Morte et al. [26].

Plant regeneration ability in adult conifers has been reported to be difficult [25]. In some cases, serial bud grafting treatments may improve the in vitro performance of these explants [24]. In our work, explants taken from adult trees had comparable results to those obtained in [26] using seedling explants and it was not necessary to apply grafting treatments to obtain active-growing shoots under in vitro conditions, thus highlighting the suitability of the procedure applied here.

DPS increases plant tissue surface area for medium components and water uptake, enhances nutrient diffusion as well as other substances required for the plant development to grow cells and tissues, and improves diffusion of waste products and growth inhibitors away from growing tissue [49]. These physical properties provided by the addition of liquid medium were found to stimulate embryogenic tissue development in vitro for other conifers such as *Pseudotsuga menziesii* (Mirb.) Franco [50] or *Pinus taeda* L. [49]. In the present work, DPS also significantly increased the elongation ability of *T. articulata* explants. The sucrose present in the liquid phase of the DPS seemed to play a key role in this shoot elongation as treatments lacking it performed significant lower morphogenic responses over the 8-week culture period. The positive role of sucrose in stimulating morphogenic responses of in vitro cultured plants has been quite well documented in several plant developmental processes, for instance, such as in the organogenesis of kohlrabi [51], bulb swelling [39,52], plantlet root induction [53], and shoot elongation [54,55]. It is still not clear whether carbohydrates act solely or synergistically as signaling molecules, sources of energy, or building blocks to influence the expression of certain genes involved in the PGRs’ metabolism during the organogenesis in vitro. Carbohydrates are certainly required as a source of energy during in vitro organogenesis due to the condition of the mixotrophy of cultured cells to supply the necessary carbon for plant growth and development [53]. Additionally, carbohydrates control the expression of several genes in plants and regulate both metabolic and developmental processes with an impact in plant in vitro morphogenesis [51]. Piqueras et al. [55] found a higher activity of invertases on *N. tabacum* cultured in a DPS containing 5% sucrose, which indicates an activation of the carbohydrate metabolism required for the plant growth and development, a high-energy consuming process [53]. Piqueras et al. [55] attributed the beneficial effects of the osmotic stress impressed by the sucrose contained in a DPS on *N. tabacum* in vitro culture as it might have decreased the water and cytokinin absorption that resulted in shoot elongation. However, in the present work, cytokinins were absent in the DPS composition and therefore the positive effect of the sucrose contained in the DPS on shoot elongation seems related to its nutritional role and ability in activating metabolic and developmental processes rather than to other factors such as the lower availability of PGRs, as was also observed in [54,56].

The efficiency of adventitious rooting is highly variable among conifers and it still constitutes one of the major challenges in their production in vitro [24]. For instance, Khamushi et al. [57] found optimal rooting performances around 10% in *Cupressus sempervirens* var. *horizontalis*. In *T. articulata*, rooting percentages obtained in [26] were of 60% in 2 mg L^−1^ NAA + 2 mg L^−1^ IBA. In the present work, this rooting performance was improved (66.7%) after a 4 h dipping of explants in liquid medium composed by SH salts plus 250 mg MES without PGR and a cultivation for 8 weeks on SH basal salts plus 30 g L^−1^ sucrose, 6.6 g L^−1^ agar, and 0.5 g L^−1^ AC. Pulse treatments consisting in a short immersion of the explants in liquid medium have been reported to be more effective in rooting than usual culture onto solid media for *C. sempervirens* var. *horizontalis* (Mill.) Loudon [57] and *Mitragyna parvifolia* (Roxb.) Korth. [58].

Genetic and epigenetic changes (such as gene amplification, mutation, chromosomal rearrangement, and retrotransposon activation) during the in vitro culture between regenerated plants and their corresponding mother plants (somaclonal variation) is often a matter of concern in biotechnology applied to the conservation of rare and threatened plant species [29,31], as well as in large-scale commercial production of economically important crop plants [30,59,60]. Therefore, it is necessary to assess the genetic stability of plants produced under in vitro conditions to ensure uniformity of the obtained plantlets within clonal propagation (in comparison to the mother plant) or to study that those changes eventually produced do not negatively impact on the range of genetic diversity and structure of natural populations in reintroduction activities [61]. RAPD markers are based on the non-coding regions of DNA and constitute a useful approach for an optimal assessment of the genetic uniformity of regenerated plantlets [59,62]. The use of RAPD markers for these proposes has been also applied recently in micropropagated, threatened plants with medicinal interests such as *Rhynchostylis retusa* (L.) Blume [63], *Prunus africana* (Hook f.) Kalkman [31], *Rhododendron mucronulatum* Turcz [64], or *Typhonium flagelliforme* (Lodd.) Blume [65]. Our results showed that the amplified products resulted in monomorphic bands in micropropagated plantlets in comparison to the mother plant. None of the selected primers showed any polymorphism in micropropagated plants, indicating the genetic stability of the in vitro cultured plants. Therefore, the approach presented here provides, for the first time, reliable information on the genetic stability of *T. articulata* plants produced under in vitro conditions. This information is very important as genetic fidelity is crucial for the proper re-introduction or reinforcement of individuals in natural populations in conservation strategies.

In the acclimatization phase, 90% of the plantlets survived over four weeks of cultivation under ex vitro conditions. Although at lower percentages of approximately 70%, Morte et al. [26] also obtained a high acclimatization success after in vitro rooting, in contrast to what is reported of other coniferous species [22,42]. The drought tolerance of *T. articulata* plants in natural conditions has been highlighted in recently published studies due to the biological characteristics of this tree. For instance, hydraulic properties related to the water-use strategies have demonstrated that *T. articulata* is very resistant to drought [66] and more resistant than other conifers such as pines [67]. As the acclimatization phase involves the ability to regulate the water management in order to face the drier environment imposed by ex vitro conditions such as lower humidity and higher light intensity, among other stressing factors [68,69], these intrinsic features of *T. articulata* plants may explain the higher acclimatization success found in this study in comparison to the previously reported results on other conifers.

In order to develop a germplasm conservation approach based on the slow-growth strategy, the effects of low temperatures in explant survival and regrowth (measured as the length increment) were evaluated for two storage periods. During the cold storage, 100% of explants treated at 10 °C for 12 weeks survived, whereas those disposed at lower temperatures and longer storage periods showed lower percentages of survival. This contrasts to what was obtained for some other woody plants, such as grapevine [70], *Camellia japonica* L., and *C. reticulata* Lindley [71] or *Quercus suber* L. [72], where optimal storage conditions were achieved at 4.0–5.0 °C during longer periods of storage (12 and 24 months) rather than at temperatures closer to 10 °C [70], as we obtained for *T. articulata*. Nonetheless, the optimal survival rates in [70,72] were between 25% and 50% lower than in the present study, notably even lower than in those explants stored at 4°C for 28 weeks. In conifers, the information on the cold storage of plantlets and tissues produced in vitro through slow-growth techniques is rather more limited than in angiosperms. In this regard, *Sequoia sempervirens* (D. Don.) Endl. showed similar results and survival rates were higher than 90% after a cold storage at 4 °C in dark conditions for 12 months [73], as we obtained in the present study. However, the treatment at 10 °C results in a more suitable temperature than 4 °C to store the explants for a medium-term conservation strategy for *T. articulata*. This approach provides an alternative and optimal method to the cryopreservation protocol by vitrification as previously described in [32]. In addition, it allows for an extension of the intervals between subcultures during the maintenance of in vitro cultures, as described in [70,71].

Finally, it is generally assumed that there can be a significant level of variability among genotypes (or ecotypes) of the same species. This fact is often related to the different response of the explants from different origins to the same in vitro culture conditions (initiation, multiplication, etc.). For this reason, this work presents the basis for the micropropagation of *T. articulata* from adult trees. However, it would be desirable to compare the culture success and propagation in wild trees from different locations in order to more accurately design a strategy of ex situ conservation based on the use of in vitro culture techniques.

## 4. Materials and Methods

### 4.1. Sterilization of the Plant Material and Culture Initiation

Actively growing twigs of *T. articulata* were collected between December and February from two healthy 19-year-old trees growing in the University of Alicante Campus (to perform the experiments aimed at the development of the in vitro culture procedure) and from a wild population located in Calblanque (Murcia, Spain; to test the somaclonal variation of in vitro produced plants). The cuttings (Figure 1A) were washed thoroughly in tap water, soaked in water with a few drops of liquid soap for 1 h, and finally rinsed with sterile distilled water. Then, the washed cuttings were surface-sterilized by immersion in 70% (*v*/*v*) ethanol (VWR Chemicals, Llinars del Vallès, Spain) for 30 s, followed by 15 min in 10% (*v*/*v*) commercial bleach (40 g L^−1^ active chlorine content) with 0.05% (*v*/*v*) Tween-20^®^ (Merck KGaA, Darmstadt, Germany). Finally, the cuttings were rinsed 5 times with sterile distilled water. Then, explants of about 20 mm in length were vertically cultured in 25 × 150 mm test tubes with polypropylene caps used to seal the tubes (Auxilab S.L., San Ginés, Spain) containing SH basal medium [74] supplemented with 30 g L^−1^ sucrose and 6.5 g L^−1^ Plant agar (Duchefa Biochemie, Haarlem, The Netherlands). Cultures were incubated in a growth chamber at 25 ± 1 °C under low active radiation conditions (0.7 μmol m^−2^ s^−1^) for 3 weeks and then placed at 42 μmol m^−2^ s^−1^ in a 16 h photoperiod of red–blue light using Gro-lux type tubes (Sylvania Lamps, Erlangen, Germany). Both sterilization and explant sowing were conducted under aseptic conditions provided by a laminar flow cabinet (Indelab S.A., Barcelona, Spain). Five independent experiments using 72–114 explants were conducted for sterilization and in vitro culture initiation. Several passages were performed on a selected explant in the medium and conditions above-described in order to obtain a stock of axenic and clonal plant cultures for the further experiments. Media and growth regulators employed in this work were purchased from Duchefa Biochemie (Haarlem, Netherlands). The pH of all media was adjusted to 5.75–5.8 with 0.1 M NaOH or 0.1 M HCl before autoclaving for 20 min at 121 °C.

### 4.2. Growth Regulator Effects on Multiplication

Twenty-one media composed by four PGRs were tested for their effects on *T. articulata* growth parameters under in vitro culture conditions. Previously initiated shoots (1.5–2 cm long and 3–5 branches) were used as the explants’ source. Explants were vertically inoculated onto basal medium SH.

In a first experiment, the SH basal medium was supplemented with 13 concentrations of PGRs: BAP (0.5–4.0 mg L^−1^), KIN (0.5–4.0 mg L^−1^), or TDZ (0.025–0.1 mg L^−1^; Table 1). In a second experiment, multiplication was tested on SH medium supplemented with 9 combinations of KIN or BAP (0.5–4.0 mg L^−1^) and 0.05 mg L^−1^ NAA (Table 2). Medium SH without PGRs served as controls.

For both experiments, the explants were cultured over 6 weeks and one subculture onto fresh medium was done at the fourth week. Finally, all cultures were transferred to SH fresh medium without PGRs for 6 more weeks. Plant developmental parameters (newly formed branches and shoots and explant length increment) were measured every 2 weeks during the 12-week period of the experiment. Forty-eight explants (16 explants per treatment × 3 replicates) were inoculated.

### 4.3. Double-Phase Culture System Effects on Shoot Elongation

The effects of a DPS on the elongation of *T. articulata* shoots were investigated in two experiments.

In the first one, the effect of an overlay of liquid medium on a solid phase was investigated. Then, 24 explants of 1.8–2.5 cm in length were vertically cultured onto SH basal medium containing 30 g L^−1^ sucrose and 6.5 g L^−1^ Plant agar (Duchefa Biochemie, Haarlem The Netherlands) in test tubes with polypropylene caps (Auxilab S.L., San Ginés, Spain). After three days, a volume of 2 mL of liquid phase was added in all test tubes for each treatment. The liquid phase consisted of ½ Knop solution [75], 50 g L^−1^ sucrose, and 3 or 6 g L^−1^ AC at pH 5.0. Twenty-four explants (8 explants × 3 replicates) were used in each treatment and cultured under the above-mentioned temperature and photoperiod conditions. The shoot elongation, measured as the increase in height, was measured after 4 and 8 weeks of treatment.

The second experiment was aimed at identifying what components of the liquid overlay influenced shoot elongation. For this, 60 explants 2–3 cm in length per treatment were inoculated onto SH basal medium containing 30 g L^−1^ sucrose and 6.5 g L^−1^ Plant agar (Duchefa Biochemie, Haarlem, The Netherlands) in test tubes with polypropylene caps (Auxilab S.L., San Ginés, Spain). After three days, a volume of 1 mL of liquid phase was added in all test tubes for each treatment. Treatments involved the use of ½ Knop solution and mixtures of ½ Knop solution plus 10 g L^−1^ or 60 g L^−1^ sucrose. A treatment with sterile water was used as the control. Replenishment of the liquid phase was performed at the 2nd and 5th week of cultivation. Shoot length increment was measured regularly over an 8-week period of culture.

### 4.4. Auxin Effects on Root Development

The rooting abilities of the explants treated with the auxins NAA, IBA, and IAA were studied in two experiments:

Experiment 1. Explants 2–2.5 cm in length were transferred to SH basal medium supplemented with 30 g L^−1^ sucrose, 6.6 g L^−1^ agar, and auxins as follows: 0 (control) and 0.5 IBA, 0.5 NAA, and 0.5 g L^−1^ IBA plus 0.25 g L^−1^ NAA. A total amount of 16 explants was studied per replicate.
Experiment 2. An auxin pulse treatment was performed on explants 2–2.5 cm in length taken from previously cultured plantlets on SH medium. The pulse treatment consisted of immersion of explants under dark conditions in liquid medium composed by SH salts, vitamins, and 250 mg of 2-(N-morpholino)ethanesulfonic acid (MES) as buffering agent to keep the culture pH between 5.75 and 5.8. Three concentrations (25, 50, and 100 mg L^−1^) of Indole Acetic Acid (IAA) at three immersion times (1, 2, and 4 h) were assayed. IAA was filter-sterilized and added to the previously described formulation after autoclaving. A sample without IAA was used as the control. After the treatment, explants were transferred to SH basal medium supplemented with 30 g L^−1^ sucrose, 6.6 g L^−1^ agar, and 0.5 g L^−1^ AC. Twenty-four explants were used per treatment and the rooting percentage was measured after 4, 6, and 8 weeks of culture.

### 4.5. Culture Media Effects on Root Development

The effects of two concentrations of SH salts (full-strength SH and ½ SH), sucrose (15 and 30 g L^−1^), and AC (0 and 0.9 g L^−1^) on rooting were also evaluated. Shoots 2–2.5 cm long were vertically cultured on 15 mL of the media solidified with 6.5 g L^−1^ plant agar (Duchefa Biochemie, Haarlem, The Netherlands). Twenty explants were used per treatment. The percentage of explants that rooted were measured and recorded over a 10-week period of culture.

### 4.6. Extraction of Genomic DNA and RAPD Analysis

To assess the genetic stability of the in vitro propagated plants, RAPD analysis was conducted on 14-month-old in vitro rooted plantlets obtained from the wild population of Calblanque, and cultured in the same conditions as the progeny obtained from the University of Alicante (PGRs:0.5 mg L^−1^ BAP plus 0.05 mg L^−1^ NAA). A sample taken from the mother plant growing in natural environments served as the control. Genomic DNA was extracted from 200 mg of fresh tissue obtained from randomly harvested branches from both the maternal plant and the in vitro regenerated plants (approximately 20 mg of a dry-cryodesiccated weight) with a DNeasy^®^ Plant Mini Kit (Qiagen, Hilden, Germany). Purified DNA was then cold stored at −20 °C before further analysis. RAPD amplification was performed in a final reaction volume of 20 μL containing a 60 ng DNA template, 100 ng of each random primer, 0.2 mM of each deoxyribonucleotide triphosphate (dNTPs), 2 μL of reaction buffer (10×), 2.5 mM of magnesium chloride (MgCl_2_), and 0.5 U of Taq polymerase (Netzyme DNA polymerase). The amplification took place using a thermal cycler Mastercycler^®^ (Eppendorf, Hamburg, Germany) consisting of the following cycle: an initial pre-heating step at 94 °C for 2 min, followed by a denaturation step consisting of 45 cycles at 94 °C for 1 min, and + 1 min at 36 °C + 2 min at 72 °C, with a final extension step consisting of 1 min at 94 °C + 1 min at 36 °C + 7 min at 72 °C. The amplification products were separated using a 2% (*w*/*v*) agarose gel containing TBE buffer at a concentration of 0.5×. Ethidium bromide at a concentration of 0.5 µg mL^−1^ was used as a dying agent. The sizes of the amplification products were determined by comparison with a 100 bp DNA ladder (Generuler Ladder, Fermentas, Waltham, MA, USA). The DNA bands in the gel were visualized under the GelDoc ultra-violet transilluminator (Biorad, Hercules, CA, USA). For photography and digitalization of the images, the software Quantity One^®^ (BioRad, Hercules, CA, USA) was used. Experiments were done twice.

### 4.7. Acclimatization

*T. articulata* microplants showing well-developed roots were taken from the tubes and gently rinsed with distilled water in order to clean the rest of the agar. Then, thirty plantlets per replicate were transferred into plastic-covered trays containing a sterilized mixture of peat moss:vermiculite (1:1) and placed in a growth chamber under the above-mentioned conditions. Relative humidity (RH) was reduced over a period of 3 weeks by progressive opening of the plastic cover. The percentage of plants that survived over the acclimatization stage was recorded after 6 weeks.

### 4.8. Medium-Term Storage of In Vitro Explants

Shoots 2–2.5 cm long obtained from stock cultures (approximately two years under in vitro culture conditions) were maintained by monthly subculture in basal medium SH. Four weeks after the last subculture, the explant length was measured and explants (one per tube) were stored at 4 °C or 10 °C in the dark. The test tubes were wrapped with a plastic layer in order to avoid medium dehydration. After 12 and 28 weeks of cold exposure (stage 1), explants were transferred to fresh SH medium and cultured at 25 ± 1 °C under an active radiation of 42 μmol m^−2^ s^−1^ in a 16 h photoperiod of red–blue light using Gro-lux type tubes (Sylvania Lamps, Erlangen, Germany) for 8 weeks (stage 2). The percentage of surviving explants and the length increase of the explants was determined by monitoring every week during the further 8-week period of cultivation. The length increase was calculated according to the equation ∆L = FL − IL, where FL is the explant length monitored during each week of stage 2 and IL the explant length before the cold exposure (stage 1).

### 4.9. Experimental Design and Data Analyses

All sets of experiments were performed with three replicates, with variable amounts of explants (stated in each section above) using a randomized block design and repeated in triplicates (with the exception of RAPD markers that were repeated twice) in order to assess the reproducibility of the results obtained. The data were analyzed statistically using one-way analyses of variance (ANOVA). For the micropropagation experiments, significant differences were determined using Duncan’s multiple range test while data regarding medium-term storage were analyzed by the T-test. Analyses were done at the 95% level of confidence using SPSS version 11.5 (SPSS Inc., Chicago, IL, USA). The results are presented as mean ± standard error.

## 5. Conclusions

An efficient approach for the in vitro propagation of *T. articulata* was developed. The SH medium supplemented with 30 g L^−1^ sucrose, 6.5 g L^−1^ plant agar, 4.0 mg L^−1^ 6-benzylaminopurine (BAP), and 0.05 mg L^−1^ NAA provided the optimum multiplication rate (90.48 ± 9.52% of explants with basal shoots and 2.58 ± 0.29 basal shoots per explant). The application of AC or the ½ Knop solution in a liquid overlay produced significantly longer shoots during an 8-week culture period. Rooting was optimal (66.7%) after a pulse treatment consisting of 4 h immersion in liquid SH medium without PGRs, followed by 8 weeks of cultivation on SH plus 30 g L^−1^ sucrose, 6.6 g L^−1^ agar, and 0.5 g L^−1^ AC. Rooted microplants were successfully acclimatized (93.33%) in a peat moss and vermiculite mixture (1:1 *v*/*v* ratio). The genetic stability of the in vitro regenerated plantlets was confirmed using the randomly amplified polymorphic DNA (RAPD) technique. Explant survival and growth remained higher than 90% after 28 weeks of cold storage at both 4 °C and 10 °C. The results presented here point out the suitability of adult-tree explants for the micropropagation and medium-term conservation of *T. articulata*.

## Figures and Tables

**Table 1:** Effect of 13 concentrations of 6-benzylaminopurine (BAP), kinetin (KIN), and thidiazuron (TDZ) on *Tetraclinis articulata* (Vahl) Masters multiplication after 6 weeks of in vitro cultivation (mean ± SE) on SH medium (Schenk and Hildebrandt, 1972) and further culture in SH medium without PGRs for 6 more weeks. Mean values within a column, followed by the same letter are not significantly different by Duncan’s multiple range test at the 95% level of confidence.

Treatment (mg L^−1^)	Length Increment (cm)		Explants with Basal Shoots (%)		Basal Shoots per Explant (nº)	
	6 Weeks	12 Weeks	6 Weeks	12 Weeks	6 Weeks	12 Weeks
BAP						
0	1.60 ± 0.21 a	2.86 ± 0.26 a	0.00 ± 00.0 c	0.00 ± 0.00 e	0.00 ± 0.00 c	0.00 ± 0.00 e
0.5	0.98 ± 0.08 bcd	2.30 ± 0.22 bc	13.81 ± 4.27 ab	53.49 ± 2.06 bc	0.32 ± 0.10 ab	1.17 ± 0.12 d
1.0	0.76 ± 0.06 cde	1.44 ± 0.10 efg	20.21 ± 4.20 a	79.85 ± 5.32 a	0.47 ± 0.19 a	2.60 ± 0.43 a
2.0	0.51 ± 0.05 def	1.26 ± 0.04 g	10.70 ± 5.58 abc	68.89 ± 4.44 a	0.24 ± 0.12 abc	1.96 ± 0.22 b
4.0	0.47 ± 0.04 ef	1.32 ± 0.09 fg	4.31 ± 2.16 cb	65.42 ± 4.97 ab	0.06 ± 0.04 bc	1.85 ± 0.05 b
KIN						
0.5	1.14 ± 0.04 b	2.71 ± 0.02 ab	0.00 ± 0.00 c	4.17 ± 2.08 e	0.00 ± 0.00 c	0.04 ± 0.02 e
1.0	1.00 ± 0.11 bc	2.18 ± 0.17 cd	0.00 ± 0.00 c	4.44 ± 2.22 e	0.00 ± 0.00 c	0.09 ± 0.06 e
2.0	0.69 ± 0.13 ef	1.89 ± 0.09 cde	8.61 ± 1.94 bc	24.4 ± 8.01 d	0.22 ± 0.04 abc	0.69 ± 0.20 cd
4.0	0.41 ± 0.04 f	1.82 ± 0.19 de	11.43 ± 2.40 ab	46.51 ± 2.06 c	0.23 ± 0.06 abc	1.19 ± 0.14 d
TDZ						
0.025	1.84 ± 0.09 a	2.89 ± 0.17 a	10.41 ± 2.08 abc	29.72 ± 3.92 d	0.19 ± 0.06 bc	0.74 ± 0.07 d
0.05	0.95 ± 0.08 bcd	1.75 ± 0.17 def	6.25 ± 3.61 bc	27.08 ± 4.17 d	0.08 ± 0.06 bc	0.48 ± 0.11 de
0.1	0.51 ± 0.11 ef	1.12 ± 0.12 gh	4.17 ± 4.17 bc	29.86 ± 9.19 d	0.06 ± 0.06 bc	0.32 ± 0.14 de
0.25	0.57 ± 0.03 ef	0.76 ± 0.06 h	4.17 ± 4.17 bc	6.25 ± 6.25 e	0.06 ± 0.06 bc	0.10 ± 0.10 e

**Table 2:** Effect of nine combinations of BAP or KIN with 1-naphthaleneacetic acid (NAA) on *Tetraclinis articulata* (Vahl) Masters multiplication after 6 weeks of in vitro cultivation (mean ± SE) on SH medium (Schenk and Hildebrandt, 1972), and further culture in SH medium without PGRs for 6 more weeks. Mean values within a column, followed by the same letter are not significantly different by Dun-can’s multiple range test at the 95% level of confidence.

Treatment (mg L^−1^)		Length Increment (cm)		Explants with Basal Shoots (%)		Basal Shoots per Explant (nº)	
		6 Weeks	12 Weeks	6 Weeks	12 Weeks	6 Weeks	12 Weeks
BAP	NAA					
0	0	2.24 ± 0.20 a	3.26 ± 0.13 a	0.00 ± 0.00 c	0.00 ± 0.00 d	0.00 ± 0.00 d	0.00 ± 0.00 e
0.5	0.05	1.28 ± 0.10 b	2.83 ± 0.20 ab	58.33 ± 8.55 a	84.29 ± 8.81 a	1.36 ± 0.19 ab	2.40 ± 0.31 ab
1.0	0.05	0.67 ± 0.04 c	1.28 ± 0.18 de	59.68 ± 13.75 a	77.38 ± 10.40 ab	1.51 ± 0.27 a	3.04 ± 0.25 a
2.0	0.05	0.42 ± 0.06 bcde	0.65 ± 0.07 ef	27.78 ± 7.73 b	75.56 ± 9.69 ab	0.62 ± 0.14 c	2.11 ± 0.09 bc
4.0	0.05	0.19 ± 0.03 e	0.38 ± 0.07 f	38.61 ± 12.55 ab	90.48 ± 9.52 a	0.86 ± 0.27 bc	2.58 ± 0.29 ab
KIN	NAA						
0.5	0.05	1.11 ± 0.04 b	3.05 ± 0.29 a	30.28 ± 12.08 ab	46.11 ± 12.03 c	0.54 ± 0.21 cd	0.99 ± 0.29 d
1.0	0.05	0.49 ± 0.02 cd	2.17 ± 0.32 bc	41.67 ± 4.16 ab	56.55 ± 3.62 bc	1.00 ± 0.19 ab	1.60 ± 0.03 c
2.0	0.05	0.41 ± 0.09 cde	1.61 ± 0.37 cd	31.75 ± 4.16 ab	66.67 ± 6.30 abc	0.75 ± 0.17 c	2.00 ± 0.18 bc
4.0	0.05	0.30 ± 0.03 de	1.35 ± 0.16 d	34.70 ± 6.10 ab	73.50 ± 5.60 ab	0.65 ± 0.08 c	2.03 ± 0.11 bc

**Table 3:** List of primers, codes, and sequences (5′-3′) of the amplified fragments generated by five out of the 31 randomly amplified polymorphic DNA (RAPD) markers employed to assess the genetic stability of *Tetraclinis articulata* (Vahl) Masters microplants.

No.	Primer Code	Primer Sequences (5′-3′)
16	BAS16	GCGATTTGCC
21	BAS21	GAGCAGCGAA
26	BAS26	CGGACGCATT
27	BAS27	ACGCTGGTAG
29	BAS29	CCACGCAACA

## Data Availability

The data produced and/or analyzed in this work are included in this published article. The datasets employed and analyzed during the present study are available from the corresponding author upon reasonable request.
